# SUMOylation at K^707^ of DGCR8 controls direct function of primary microRNA

**DOI:** 10.1093/nar/gkv741

**Published:** 2015-07-21

**Authors:** Changhong Zhu, Cheng Chen, Jian Huang, Hailong Zhang, Xian Zhao, Rong Deng, Jinzhuo Dou, Hui Jin, Ran Chen, Ming Xu, Qin Chen, Yanli Wang, Jianxiu Yu

**Affiliations:** 1 Department of Biochemistry and Molecular Cell Biology, Shanghai Key Laboratory of Tumor Microenvironment and Inflammation, Shanghai Jiao Tong University School of Medicine, 280 South Chongqing Road, Shanghai 200025, China; 2 State Key Laboratory of Oncogenes and Related Genes, Shanghai Jiao Tong University School of Medicine, Shanghai 200025, China

## Abstract

DGCR8 (DiGeorge syndrome critical region gene 8) is essential for primary microRNA (pri-miRNA) processing in the cell nucleus. It specifically combines with Drosha, a nuclear RNase III enzyme, to form the Microprocessor complex (MC) that cleaves pri-miRNA to precursor miRNA (pre-miRNA), which is further processed to mature miRNA by Dicer, a cytoplasmic RNase III enzyme. Increasing evidences suggest that pri-/pre-miRNAs have direct functions in regulation of gene expression, however the underlying mechanism how it is fine-tuned remains unclear. Here we find that DGCR8 is modified by SUMO1 at the major site K^707^, which can be promoted by its ERK-activated phosphorylation. SUMOylation of DGCR8 enhances the protein stability by preventing the degradation via the ubiquitin proteasome pathway. More importantly, SUMOylation of DGCR8 does not alter its association with Drosha, the MC activity and miRNA biogenesis, but rather influences its affinity with pri-miRNAs. This altered affinity of DGCR8 with pri-miRNAs seems to control the direct functions of pri-miRNAs in recognition and repression of the target mRNAs, which is evidently linked to the DGCR8 function in regulation of tumorigenesis and cell migration. Collectively, our data suggest a novel mechanism that SUMOylation of DGCR8 controls direct functions of pri-miRNAs in gene silencing.

## INTRODUCTION

The microRNA (miRNA) biogenesis pathway has been thoroughly uncovered. A long primary transcript known as a pri-miRNA in the cell nucleus is cleaved by a Microprocessor complex (MC), which is mainly composed of Drosha, an RNase III enzyme and DGCR8, a double-stranded RNA-binding protein (1–4), to generate a characteristic stem-loop structure of about 70 bp long, known as a pre-miRNA. The latter molecule is subsequently exported by exportin-5 to the cytoplasm and further cleaved into an ∼20–25-bp double-stranded RNA fragment by another RNAIII enzyme Dicer. Then one strand of the duplex, as a mature miRNA, is incorporated into an effector complex called the RNA induced silencing complex (RISC) composed of Ago2 together with related proteins, while the remaining strand is degraded as a substrate of RISC complex.

miRNA regulates gene expression in a negative manner by influencing the stability or the translational efficiency of target mRNAs, which is generally considered to be due to the active mature miRNA. But interestingly, increasing evidences suggest pri-/pre-miRNAs have direct functions in regulation of gene expression. Chen's group has first reported that the different activities of miR-181a-1 and miR-181c, which are members of the same miRNA gene family, are dependent on their pre-miRNA loop nucleotides other than nucleotide difference in their mature miRNA sequences (5). Later they found that pri-let-7 can directly interact with target mRNAs to show a direct function in target repression, whose activity is determined on loop nucleotides by modulating interactions between pri-let-7 and target mRNAs (6,7). In accordance with the above findings, Kay's group has also reported that pri-/pre-miR-151 directly regulates the E2f6 mRNA level by binding to its 3′-untranslated region (3′-UTR) (8). Thus, it has become increasingly clear that pri-/pre-miRNAs can serve as post-transcriptional regulators of miRNA activity besides as biogenesis intermediates.

DGCR8 gene is first discovered in the DiGeorge syndrome chromosomal region on human chromosome 22 (9). As the most important partner of Drosha, DGCR8 binds with pri-miRNA via its two double-stranded RNA-binding domains (dsRBDs) to stabilize it for processing by Drosha, which releases hairpin-structured pre-miRNA (1,2,10,11). The abnormal expression of DGCR8 accompanying with disordered miRNA biogenesis has been discovered in diverse diseases, such as cancers and schizophrenia (12–19). Recently, it has been reported that post-translational modifications (PTMs) of DGCR8 modulate in its function in miRNA biogenesis. For example, phosphorylation of DGCR8 N-terminal by MAPK/ERK pathway increases its protein stability (20) and deacetylation of DGCR8 dsRBDs by HDAC1 enhances its affinity with pri-miRNAs (21).

In this study, we found that DGCR8 was modified at the major site K^707^ by SUMO1, a small ubiquitin-like modifier, which can reversibly modulate its targets in many aspects such as activity, stability, localization and interaction with other proteins (22). Although K^707^-SUMOylation of DGCR8 did not influence the MC activity and the production of mature miRNAs, it could enhance the protein stability and the affinity of pri-miRNA with DGCR8, which controlled direct functions of pri-miRNAs in recognition and repression of the target mRNAs. Moreover, SUMOylation at K^707^ of DGCR8 was involved in the regulation of tumorigenesis and tumor cell migration, which was probably contributed to its influencing on the formation of pri-miRNA /target mRNA complex.

## MATERIALS AND METHODS

### Cell cultures and transfections

Human embryonic kidney 293T, 293FT, HeLa, A549*^luc^* cells and *SENP1*^−/-^ Mouse Embryo Fibroblast (MEFs) were cultured in Dulbecco's modified Eagle's medium (Hyclone) containing 10% Fetal Bovine Serum (FBS) (Biowest) at 37°C with 5% CO_2_. PC3*^luc^* cells that express a firefly luciferase used for living imaging (23) were cultured in RPMI1640 (Hyclone) containing 10% FBS. *SENP1*^−/−^ MEFs from *SENP1* homozygous null mice were provided by Dr JK Cheng at Shanghai Jiao Tong University School of Medicine. All transfections were performed using lipofectamine2000 (Invitrogen).

### Reagents and antibodies

Monoclonal anti-Flag M2 (#F1804) was from sigma. Monoclonal anti-HA (#A488–101L) was from Covance. Antibodies to SUMO1 (#4930), Drosha (#3364), GFP (#2555), Myc (#2278), Dicer (#3363), ZEB1 (#D80D3), p44/42 (#137F5), Phospho-P44/42-Erk1/2 (#4370) were from Cell Signaling Technology. Monoclonal anti-DGCR8 (#60084–1-Ig) and polyclonal anti-DGCR8 (#10996–1-AP), anti-β-actin (#60008–1-Ig), anti-GAPDH (#60004–1-Ig), anti-Tubulin (#66031–1-Ig) were from Protein Tech Group. Peroxidase-conjugated Affinipure goat anti mouse/rabbit IgG was from Jackson ImmunoResearch Laboratory. For western blotting analysis, anti-β-actin, anti-GAPDH and Peroxidase-conjugated Affinipure goat anti mouse or rabbit were used at a 1/5000 dilution, other primary antibodies were used at a 1/1000 dilution. Normal rabbit IgG sc-2027 (#I2310) and normal mouse IgG sc-2025 (#J1810) were from Santa Cruz Biotechnology. Protein G plus/ protein A agrose suspension (#IP05) and Recombinant Human Epidermal Growth Factor (rHu EGF) were purchased from Calbiochem. Ni-NTA beads was from QIAGEN. MG132, cyocheximide (CHX), puromycin, U0126 were purchased from Sigma.

### Plasmids

The Flag-pck-Drosha and Flag-pck-DGCR8 expression constructs were kindly provided by Dr V. Narry Kim at the Seoul National University. Point mutations of DGCR8 were carried out by using KOD-plus-mutagenesis Kit (TOYOBO) according to the procedure. The human DGCR8 full-length cDNA and mutants were amplified by using polymerase chain reaction (PCR) Master Mixture (Fermentas, #K0171) and sub-cloned into EcoRI and NotI sites of the vector pCMV-Myc (Clontech) for expression in mammalian cells and the vector pGEX4T1 for GST-fusion expression in bacteria, respectively. The Myc-DGCR8 cDNA was amplified by PCR from pCMV-Myc-DGCR8 and then was subcloned into XbalI and NotI on the lentiviral vector (System Biosciences) carrying *EGFP* and *Puromycin*. SUMO2/3 is tagged with the amino acid sequence RGSHHHHHH, termed ‘RH’ tag (24); while SUMO1 is tagged with the amino acid sequence HHHHHH, termed ‘His’ tag. Both ‘RH’ and ‘His’ tagged SUMO proteins can be identically detected by anti-His antibody. The shRNA oligonucleotides for DGCR8 /Senp1 referred from Sigma were sub-cloned into AgeI and EcoRI sites of the vector pLKO.1, respectively. Pri-miRNAs were cloned into the lentiviral vector or the psiCHECK vector by using PCR method, in which the human genomic DNA was used as a template. The sequences of all plasmids were verified by sequencing. Primer sequences used for construction are given in Supplementary Table S1.

### SUMOylation assays

- *Ni^2+^-NTA pull down assay*. 293T or HeLa cells co-transfected with Flag-DGCR8 WT/mutants and His-tagged SUMO1 together with or without Ubc9 or Senp1 were lysed and then pulled down with Ni^2+^-NTA resin as previously described (25).
- *SUMOylation assay in bacterial reconstitution system* was performed as previously described (26). Briefly, *Escherichia coli* BL21 transformed with GST-DGCR8-WT/K^707^R alone or together with pT-E1E2-SUMO1 (pE1E2S1) were cultured in the presence of 0.2 mM IPTG at 16°C overnight, then cells were harvested and lysed in the bacterial protein extraction buffer (Thermo, #PD199700) for 1 h, followed by incubation with GST-beads (GE Healthcare) at 4°C overnight, then the beads were washed with phosphate buffered saline (PBS) for three times and eluted with 500 μl GSH buffer (50 mM Tris PH 8.0, 10–20 mM GSH) for 30 min at room temperature, finally the elution was concentrated to volume of 200 μl using the Ultra-4 centrifugal filters (Millipore, Ultracel-10K). The concentration of the purified protein was measured by Bicinchoninic Acid (BCA) method and the equal amount of proteins were subjected to sodium dodecyl sulphate-polyacrylamide gel electrophoresis and blotted with antibodies against SUMO1/DGCR8.
- *SUMOylation analysis by immunoprecipitation (IP)*. For SUMO1 modification of DGCR8, PC3, A549, 293T cells or *SENP1^−/−^* MEFs and 293T cells co-transfected with Flag-DGCR8 WT/K^707^R together with or without GFP-SUMO1 were collected and washed with NEM-PBS buffer (20 mM N-ethylmaleimide in PBS) and then the cell pellets were directly lysed in NEM-RIPA buffer (50 mM Tris–HCl pH 7.4, 150 mM NaCl, 1% NP-40, 20 mM N-ethylmaleimide and one complete protease inhibitor cocktail). One milligram of total extracted proteins were used for immunoprecipitation. To detect the endogenous SUMO1-DGCR8 in PC3 or 293T cells or *SENP1^−/−^* MEFs, 5 μl of DGCR8 antibody or normal IgG (as a control) was used for immunoprecipitation, and followed by immunoblotting with anti-SUMO1 antibody. Lysates from 293T cells were incubated with anti-Flag antibody and then anti-GFP antibody were blotted.

### Ubiquitination analysis by IP

For ubiquitination analysis of DGCR8, HeLa cells co-transfected with Flag-DGCR8 WT/K^707^R together with or without Myc-Ub plasmid were lysed in RIPA buffer (50 mM Tris–HCl pH 7.4, 150 mM NaCl, 1% NP-40, and one complete protease inhibitor cocktail). One milligram of total extracted proteins were used for immunoprecipitation with anti-Flag antibody and immunoblotted with anti-Myc antibody.

### Co-immunoprecipitation

293T/HeLa cells transfected with Flag-DGCR8-WT or -K^707^R were lysed in RIPA buffer. Five hundred micrograms of total extracted proteins were incubated with 30 μl of protein A/G agarose and anti-Flag antibody, and then whirled on an vertical roller at 4°C overnight. The immunoprecipitated complexes were washed with RIPA buffer for three times and followed by western blotting analysis.

### qRT-PCR analysis

Total RNAs were firstly extracted from cells with Trizol reagent (Invitrogen), then 1μg of total RNAs were treated with DNaseI (Thermo, #EN0521) to degrade the genomic DNA. Reverse transcription was performed by using Avian Myelobastosis Virus (AMV) reverse transcriptase (Takara, #RR037A). For mature miRNAs, the miRNA-specific primers and U6 reverse primer were simultaneously added; for pri-miRNAs, random primers were utilized according to the manufacturer's instruction. qPCR was performed by using SYBR Green Master PCR Master Mix (Applied Biosystem) on StepOnePlus Real-Time PCR System (Applied Biosystem). Data were normalized to an endogenous U6 or GAPDH and quantified with the 2-ΔΔCt method.

### RNA immunoprecipitation assay (RIP)

The RNA immunoprecipitation assay (RIP) was performed as previously described (27). Briefly, 48 h after transfection with the indicated plasmids, 293T cells cultured in 10-cm plates were lysed in RIP buffer (50 mM Tris, 150 mM NaCl, 10 mM EDTA, 5 mM MgCl2, 1 mM DTT, 100 Units RNAse inhibitor (#E00381), 400 μM Ribonucleotide Vanadyl Complex (RVC) (NEB, #S1402) and one complete protease inhibitor cocktail). Then one-tenth of lysates were reserved as the input for qRT-PCR analysis, while 1/50 of lysates were saved for western blotting to examine the expression of DGCR8. Then the remaining lysates were incubated with 40 μl of protein A/G agarose along with 4 μg of anti-Flag antibody at 4°C overnight. The immunoprecipitated complex was washed with RIPA buffer for three times and 1/20 of the beads were taken out for western blotting analysis of the efficiency of immunoprecipition, the others were treated with Trizol and subjected to qRT-PCR for pri-miR130b.

### The reporter assay for the MC (Microprocessor complex) activity

The activity of MC was determined according to the method described previously (28). Briefly, the pri-miR130b containing pre-miR130b (stem-loop structure) with the flanking upstream and downstream sequences was inserted into the 3′-UTR of Renilla luciferase gene with XhoI and NotI in the psiCHECK2 vector thereby getting the microprocessor reporter construct psiCHECK-pri-miR130b (Supplementary Figure S4B). A total of 200 ng of psiCHECK-pri-miR130b with or without equal amount of Flag-DGCR8-WT/K707R and Drosha were co-transfected into 293T or HeLa-shDGCR8 cells by lipofectamine 2000. After transfection for 36 h, cells were rinsed by PBS and lysed with 100 μl of 1× Passive Lysis Buffer (PLB) for 15 min at room temperature and then 20 μl of the lysate were transferred to the multi-well plate for the dual luciferase reporter assay (Promega). For measurements, injectors of both LAR II and Stop & Glo^®^ Reagent were set as 100-μl and a 2-s delay with 24-s read time was used. Finally, the microprocessor activity was calculated by normalization with the Firefly luciferase to Renilla luciferase.

### The reporter assay for pri-let-7a activation

The four-repeated sequence complementary to let-7a-3 seed sequence (4 × let-7a-3 complementary sequences (LCS)) was inserted into the 3′-UTR of Renilla luciferase in the psiCHECK2 vector thus generating the pri-let-7a-3 activation reporter psiCHECK2–4LCS (Supplementary Figure S5D). Then pri-let7a-3 and psiCHECK2–4LCS together with or without DGCR8-WT or DGCR8-K^707^R were transfected to 293T-shSenp1 cells. Forty-eight hours after transfection, cells were lysed and subjected to the dual luciferase reporter assay according to the instruction. The activation of pri-let7a-3 by DGCR8 was calculated by normalization with the Firefly luciferase to Renilla luciferase.

### Soft agar colony formation assays

The effect of DGCR8-WT or DGCR8-K^707^R on anchorage-independent growth was determined by a soft agar assay as described previously (23). Briefly, this assay was performed in 6-well plates with a base of 2 ml of medium containing 1% FBS with 0.6% Bacto agar (Amresco). Cells were seeded in 2 ml of medium containing 1% FBS with 0.35% agar at 1.5 × 10^3^ (for PC3 cells) or 2 × 10^3^ (for A549 cells) cells/well and layered onto the base. The colonies were stained with 0.05% crystal violet at day 21 (for PC3) or 14 (for A549), and then photographs were taken and the number of colonies was scored by ImageJ V1.45 (NIH, USA). Three independent experiments were performed in triplicate.

### Proliferation assay by RTCA-DP

The E-Plate 16 was pre-equilibrated with 100 μl of medium in the tissue culture hood for 0.5–1 h and then 2 × 10^3^ of cells resuspended in 100 μl of medium were added, and kept in the culture hood for another 30 min. The real-time recording of proliferation was carried out on the RTCA-DP instrument (Roche) and monitored every 1 h for 3 days.

### Migration assay by RTCA-DP

The method was carried out as described previously (29,30). Briefly, PC3 or A549 cells were pre-treated with serum-free medium for 6 h and then 2 × 10^4^ of cells resuspended in 100 μl of serum-free medium were added into the pre-equilibrated upper chambers of the CIM-plate. The lower chamber was filled with 160 μl of normal growth medium containing 10% FBS. The kinetic cell indexes of their migration were recorded every 15 min for 3 days.

### Mouse xenograft models

Murine xenografts models were established as described previously (23). Briefly, 6-week-old nude mice were subcutaneously injected in the back with 100 μl of medium containing 2.5 × 10^6^ of PC3*^luc^* or A549*^luc^* cells stably expressing DGCR8-WT or DGCR8-K^707^R. Fourteen days after injection, tumors were measured with the IVIS system (Xenogen, Alameda, CA, USA). All mice were sacrificed at 30 days and tumors were dissected, photographed and weighted. All animal studies were conducted with the approval and guidance of shanghai Jiao Tong University Medical Animal Ethics Committees.

### Statistical analysis

All data are presented as means ± standard deviation (SD) for western blotting or means ± standard error of the mean (SEM) for the dual luciferase reporter assay, qPCR, real-time cell analysis (RTCA) migration, mouse xenograft model and soft agar colony assay. Statistical calculations were performed with Microsoft Excel analysis tools. Differences between individual groups are analyzed using the *t*-test (two-tailed and unpaired). A *P*-value of < 0.05 (*), < 0.01 (**) or < 0.001 (***) is considered significant.

## RESULTS

### DGCR8 is modified by SUMO1 in cells

**Figure 1.**
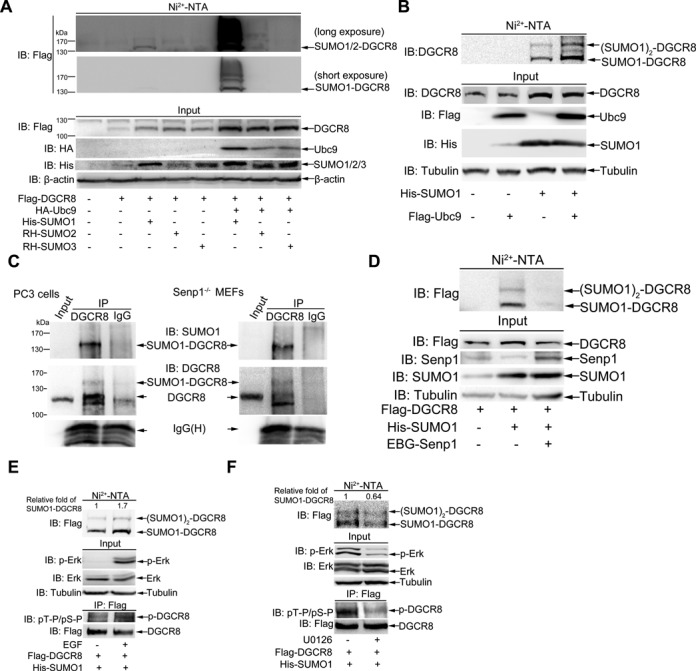
DGCR8 is modified by SUMO1. (**A**) DGCR8 is modified mainly by SUMO1. 293T cells were co-transfected with Flag-DGCR8 and three SUMO isoforms along with or without HA-Ubc9. Forty-eight hours after transfection, cells were lysed and pulled down with Ni^2+^-NTA resin for SUMOylation assay, and SUMOylated modification of DGCR8 was detected with anti-Flag antibody. (**B**) Endogenous DGCR8 is modified by SUMO1 at multiple sites. His-SUMO with or without Flag-Ubc9 were co-transfected into 293T cells and the SUMOylation assay were conducted with the method of Ni^2+^-NTA resin. (**C**) SUMOylation of DGCR8 occurs naturally in cells. PC3 cells of or *Senp1*^−/−^ MEFs were directly lysed in NEM-RIPA buffer, then immunoprecipitated complexes with anti-DGCR8 or normal IgG were immunoblotted with anti-SUMO1 antibody, and the same membrane was stripped for immunoblotting with anti-DGCR8 antibody. One-tenth of lysates as an input were immunoblotted. (**D**) SUMOylation of DGCR8 can be removed by Senp1. Flag-DGCR8 with or without His-SUMO1 or EBG-Senp1 plasmids were transfected into 293T cells. The SUMOylation assay with Ni^2+^-NTA resin was performed. (**E** and **F**) SUMOylation of DGCR8 is enhanced by EGF. Flag-DGCR8 and His-SUMO1 were transfected into 293T cells. (E) Twenty-four hours after transfection cells were incubated in serum-free medium for 24 h, then EGF (200 μg/ml) was added for 5 min. (F) Forty-eight hours after transfection cells were treated with U0126 (10 μM) for 1 h. Half of the cells were used for the SUMOylation assay with Ni^2+^-NTA resin (upper panels), while others lysed with RIPA buffer were for IP with anti-Flag antibody and followed by immunoblotting with pT-P/pS-P antibody (low panels). One-tenth of lysates in RIPA buffer as input were detected with anti-pERK, total ERK antibodies (middle panels). The relative fold of SUMO1-DGCR8 was analyzed by ImageJ (V1.45).

We analyzed the possible SUMOylation sites of DGCR8 by SUMOplot (http://www.abgent.com/sumoplot) (Supplementary Figure S1A) and found some potential consensus motifs with high scores. To determine whether DGCR8 can be truly SUMOylated, we transfected Flag-DGCR8 together with 6xHis-SUMO1, or RH-SUMO2 or RH-SUMO3, and HA-Ubc9 (SUMO E2-conjugating enzyme) into 293T cells. The His/RH-tagged SUMO conjugates pulled down with Ni^2+^-NTA agarose beads as described before (23,25) were immunoblotted. As shown in Figure 1A, DGCR8 was strongly modified by SUMO1 but very weakly by SUMO2/3. To confirm whether endogenous DGCR8 can be SUMOylated by SUMO1, 293T cells were transfected with His-SUMO1 or Flag-Ubc9 or both plasmids. The relative molecular weight (MW) of DGCR8 shifted from 115 to 135 kDa was observed in the presence of His-SUMO1 alone, and the 135- and 155-kDa bands were greatly strengthened with additional plasmid Flag-Ubc9 (Figure 1B), indicating that DGCR8 can be modified by SUMO1 at multiple sites. To further prove that SUMO1 modification of DGCR8 occurs naturally in cells, *Senp1*^−/-^ MEFs, PC3 and 293T cells were lysed in NEM-RIPA buffer and immunoprecipitated with anti-DGCR8 antibody, followed by immunoblotting with anti-SUMO1 antibody. In these three cell types, one band in a size about Mr ∼135 kDa appeared only in the immunoprecipitated complexes with anti-DGCR8 antibody but not with normal IgG (Figure 1C, Supplementary Figure S1B). Moreover, SUMO1-DGCR8 bands with the sizes of 135 and 155 kDa were greatly weakened by the expression of Senp1, which is a de-SUMOylation enzyme (31) (Figure 1D). Taken together, above data demonstrate that DGCR8 is modified by SUMO1 in cells.

### SUMOylation of DGCR8 is promoted by its phosphorylation

Since there are some cross-talks among PTMs (32) and DGCR8 can be phosphorylated by ERK/MAPK (20), there may be a relationship between SUMOylation and phosphorylation of DGCR8. As expected, DGCR8 was highly phosphorylated by activated ERK (p-ERK) with stimulation of EGF (Figure 1E, IP and Input panels), and SUMOylation of DGCR8 was enhanced in parallel (Figure 1E, Ni^2+^-NTA panel). On the contrary, the phosphorylation levels of both ERK and DGCR8 were decreased by treatment with U0126, an inhibitor for MEK1/2 and DGCR8 SUMOylation was also reduced expectedly (Figure 1F). Moreover, four phospho-sites S109, S153, T371 and S377 of DGCR8, which are the best potential phosphorylation sites by ERK/MAPKs kinase according to the GPS software (Prediction of Kinase-specific Phosphorylation Sites, V2.1.2), were simultaneously mutated as a mutant S109V/S153V/T371A/S377V, named as DGCR8-Mut4. Indeed, the SUMOylation level of DGCR8-Mut4 was greatly reduced compared to that of DGCR8-WT (Supplementary Figure S1C). These data indicate that SUMOylation of DGCR8 can be promoted by its phosphorylation.

### K^707^ is a major SUMO-Site of DGCR8

**Figure 2.**
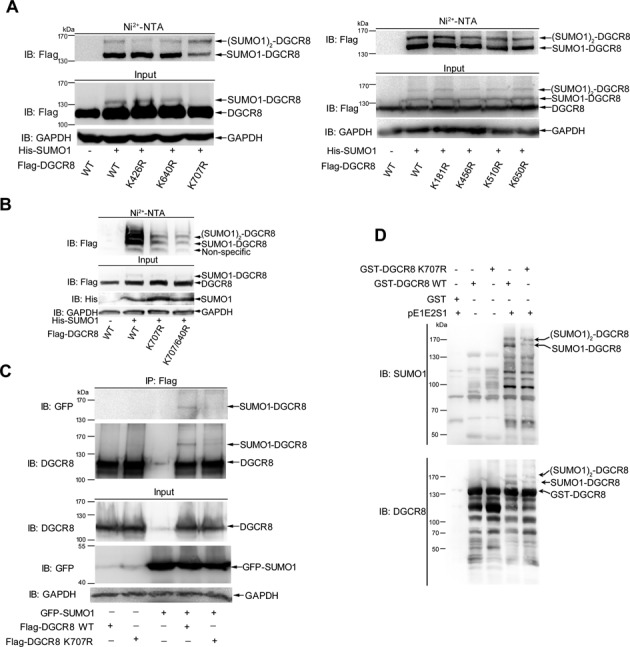
(**A** and **B**) K^707^ is a major SUMO-site of DGCR8. 293T cells co-tranfected with DGCR8 WT or mutants K^426^R, K^640^R, K^707^R, K^181^R, K^456^R, K^510^R, K^650^R or K^707/640^R with or without His-SUMO1 were lysed for the SUMOylation assay with Ni^2+^-NTA resin. (**C**) SUMOylation at K^707^ of DGCR8 confirmed by IP method. 293T cells transfected with Flag-DGCR8-WT or -K^707^R with or without GFP-SUMO1 were lysed in NEM-RIPA buffer for immunoprecipitation with anti-Flag antibody and then immunoblotting with anti-GFP antibody. The same membrane was detected with anti-DGCR8 antibody after stripping. One-tenth of lysates as input were analyzed with indicated antibodies. (**D**) SUMOylation of DGCR8 at K^707^ is verified by *in vitro Escherichia coli*-based SUMOylation reconstitution assay. *E*. *coli* BL21 cells transformed with GST-DGCR8-WT or -K^707^R and pE1E2SUMO1 were cultured at 16°C in the presence of 0.2 mM IPTG. GST-DGCR8 proteins were purified and analyzed by immunoblotting with anti-SUMO1 (upper panel) or anti-DGCR8 (low panel) antibody.

To determine which lysines (Ks) of DGCR8 are truly SUMOylated, we generated a series of DGCR8 mutants with R (arginine) replacing K according to the SUMOplot predication (Supplementary Figure S1A). 293T cells co-transfected the plasmid His-SUMO1 with Flag-DGCR8 wild-type (WT) or mutant constructs were lysed at 48 h after transfection for the SUMOylation assay with Ni^2+^-NTA resin. Compared with WT, K^426^R, K^640^R, K^181^R, K^456^R, K^510^R and k^650^R, the mutation of K^707^R most notably reduced the levels of SUMO1-DGCR8, although it did not completely remove the two bands of SUMO1-DGCR8 and (SUMO1)_2_-DGCR8, which were covalently conjugated with one and two molecule of SUMO1, respectively (Figure 2A). Therefore, we further compared DGCR8 SUMOylation of the double KR-mutant K^640/707^R to that of the single KR-mutant K^707^R and found that SUMO1 modification of DGCR8-K^640/707^R still had two bands, although showing weaker than those of DGCR8-K^707^R (Figure 2B). These data suggest that K^707^ of DGCR8 is a potential major SUMOylation site. To further confirm this, we co-transfected Flag-DGCR8-WT or -DGCR8-K^707^R with or without GFP-SUMO1 into 293T cells, and then performed immunoprecipitation with anti-Flag antibody and immunoblotting with anti-GFP antibody. The result showed that Flag-DGCR8-WT but not -DGCR8-K^707^R was SUMOylated with a shifted band in a size of MW ∼135 kDa (Figure 2C). Moreover, an *in vitro E. coli*-based SUMOylation reconstitution assay with pE1E2SUMO1 (26,33) was also carried out to verify this SUMOylation site. The results of immunoblotting with either anti-SUMO1 or anti-DGCR8 antibody showed that GST-DGCR8-K^707^R was less SUMOylated compared to those of GST-DGCR8-WT (Figure 2D). In addition, A549 cell lines stably expressing DGCR8-WT or DGCR8-K^707^R were used for detection of SUMO1 and SUMO2/3 modification of DGCR8 with the immunoprecipitation method. The results showed that SUMOylation of DGCR-K^707^R mutant was greatly reduced compared to that of DGCR8-WT (Supplementary Figure S2, the second panel in IP), whereas SUMO2/3 modifications were very faint in both DGCR8-WT and DGCR8-K^707^R mutants as expected (top panel in IP). Collectively, above results suggest that K^707^ is indeed a major SUMOylation site of DGCR8.

### SUMO1 modification of DGCR8 enhances its protein stability by blocking ubiquitination

**Figure 3.**
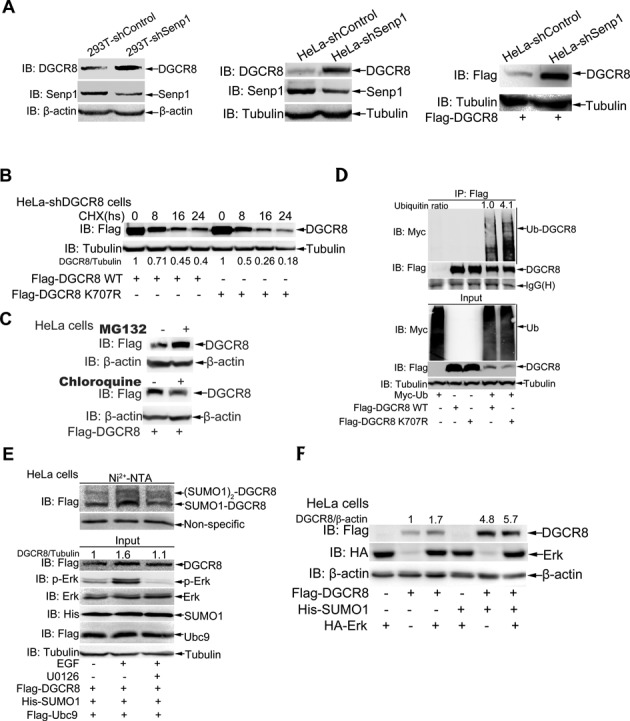
SUMOylation of DGCR8 enhances its protein stability. (**A**) Either endogenous or ectopic DGCR8 is more stabilized in Senp1 knockdown 293T or HeLa cells. Indicated 293T and HeLa-shControl or -shSenp1 cells transfected with or without Flag-DGCR8 were lysed in SDS-lysis buffer and then subjected to western blotting for detection of the protein level of endogenous DGCR8 or Flag-DGCR8. (**B**) Half-life of DGCR8-K^707^R is shorter than that of DGCR8-WT. HeLa-shDGCR8 cells were transfected with Flag-DGCR8-WT or K^707^R. Twenty-four hours after transfection cells were treated with 100 μg/ml cycloheximide (CHX) as indicated time. Cells were lysed for immunoblotting analysis. Quantification was analyzed by ImageJ (V1.45) and the Flag-DGCR8 bands were normalized with the Tubulin bands. (**C**) DGCR8 degrades mainly through the proteasome pathway. HeLa cells were transfected with Flag-DGCR8, 36 h after transfection cells were treated with 40 μM MG132 or 100 μM chloroquine for 6 h and then harvested for immunoblotting analysis. (**D**) The ubiquitination level of DGCR8-K^707^R is higher than that of DGCR8-WT. Flag-DGCR8-WT or -K^707^R with or without Myc-ub were transfected into HeLa cells, 48 h after transfection cells were lysed in RIPA buffer and immunoprecipited with anti-Flag antibody, followed by immunoblotting with anti-Myc antibody. One-tenth of lysates as input were analyzed with indicated antibodies. The relative ratios of all ubiquitin bands of lane 4 and lane 5 in IP was analyzed by ImageJ (V1.45). (**E**) The protein level of DGCR8 is paralleled with its SUMOylation levels. Flag-DGCR8, His-SUMO1 and Flag-Ubc9 were co-transfected into HeLa cells, 24 h after transfection cells were incubated in serum-free medium for 24 h, then 10 μM U0126 was added for 1 h and followed with treatment of 200 μg/ml EGF for 5 min before harvesting. Cells were lysed for the SUMOylation assay with Ni^2+^-NTA resin. (**F**) DGCR8 can be synergistically stabilized by phosphylation and SUMOylation. Flag-DGCR8 along with or without His-SUMO1 and HA-Erk were transfected into HeLa cells, 48 h later, cells were harvested by SDS buffer.

SUMO1 modification usually does not directly mediate the degradation of its substrates through the proteasome pathway, but in contrast some targets such as Smad4 (34), PCNA (35) can be stabilized by SUMOylation. Thus, we wondered whether SUMO1 modification of DGCR8 affects its protein ability. Firstly, we showed that the expression level of endogenous DGCR8 was significantly increased in the stable Senp1-knockdown 293T or HeLa cells when compared to that in the shControl infected cells (Figure 3A, left and middle panels). Meanwhile, we also found that the protein level of exogenous Flag-DGCR8 expressed in the stable Senp1-knockdown HeLa cells was much higher than that in the shControl transfected cells (Figure 3A, right panels). These data imply that SUMOylation potentially stabilizes DGCR8 protein. To confirm this, we then checked the half-life of endogenous DGCR8 proteins in HeLa cells transfected with HA-SUMO1 or control plasmid by a chase assay of CHX treatment, and found that the half-life of DGCR8 was prolonged in cells transfected with HA-SUMO1 (Supplementary Figure S3A). Next we wondered whether the half-life of DGCR8-K^707^R is shortened. To exclude the potential interference from endogenous DGCR8, a stable HeLa-shDGCR8 cell line was generated with the lenti-viral vector system carrying DGCR8 shRNA targeted to its 3′-UTR region (Supplementary Figure S3B). Then we compared the protein levels of DGCR8-WT and DGCR8-K^707^R transiently re-expressed in HeLa-shDGCR8 cells (Supplementary Figure S3C) and found that the half-life of DGCR8 was shortened from 10.1 to 8.3 h when its K^707^ mutated into R (Figure 3B). These results suggest that SUMO1 modification at K^707^ of DGCR8 indeed enhances its protein stability.

To test whether DGCR8 degradation is mainly depend on the proteasomal or lysosomal pathway, we treated HeLa cells with MG132, a proteasome inhibitor or chloroquine, a lysosome inhibitor, respectively. The result showed that DGCR8 protein was accumulated in cells treated with MG132 but not with chloroquine (Figure 3C), which revealed that DGCR8 is mainly degraded through the ubiquitin proteasome pathway. Based on above data, we questioned the increased stability of DGCR8 by SUMOylation is contributed to this modification inhibiting ubiquitination. To confirm this, we transfected Flag-DGCR8-WT/-K^707^R with or without Myc-Ub into HeLa cells. Cell lysates were used for immunoprecipitation with anti-Flag antibody and followed by immunoblotting analysis, showing that the ubiquitination of the mutant DGCR8-K^707^R was strengthened compared to that of DGCR8-WT (Figure 3D). Above data suggest that SUMO1 modification of DGCR8 enhances its stability by blocking ubiquitination.

Since our data demonstrated that phosphorylation of DGCR8 promotes its SUMOylation (Figure 1E, F and Supplementary Figure S1C), we wondered whether the crosstalk of these two modifications can synergistically stabilize DGCR8 protein. To further verify this, HeLa cells were transfected with plasmids Flag-DGCR8, Flag-Ubc9 and His-SUMO1. Compared to the vehicle treatment, the protein level of DGCR8 was 1.6-fold higher along with expectedly enhanced SUMOylation of DGCR8 by treatment with EGF. With pre-treatment of U0126 prior to EGF, the full increase of DGCR8 SUMOylation induced by EGF was inhibited and consistently the protein level of DGCR8 went down to 1.1-fold (Figure 3E). Furthermore, Flag-DGCR8 were co-transfected with either HA-ERK for activating phosphorylation or His-SUMO1 for activating SUMOylation or both plasmids into HeLa cells. Immunoblotting results showed that the protein levels of DGCR8 were increased about 1.7-, 4.8- and 5.7-fold by phosphorylation, SUMOylation and these both modifications, respectively (Figure 3F), which suggests that DGCR8 can be synergistically stabilized by phosphylation and SUMOylation, and the latter is more efficiently than the former. Taken together, our data demonstrate that SUMOylation of DGCR8 promoted by its phosphorylation enhances its stability by blocking ubiquitination.

### SUMOylation at K^707^ of DGCR8 slightly affects miRNA biogenesis and the microprocessor activity

**Figure 4.**
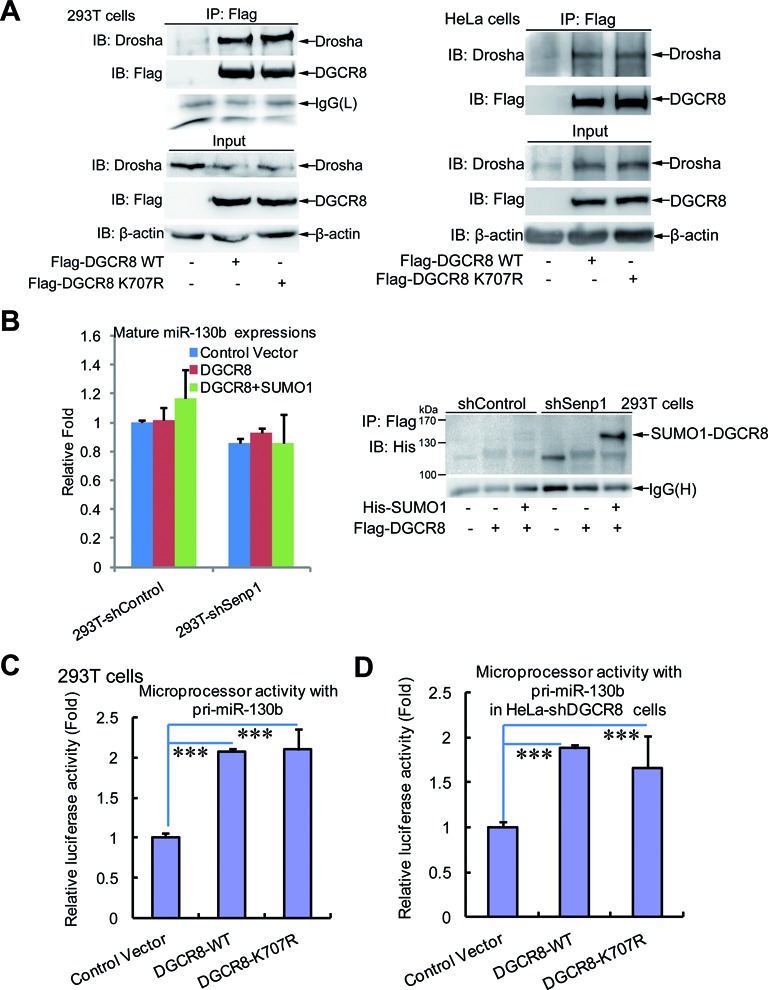
SUMOylation of DGCR8 has little effect on the microprocessor activity and miRNA biogenesis. (**A**) The SUMO-site mutation K^707^R of DGCR8 does not alter its interaction with Drosha. Lysates from 293T or HeLa cells transfected with Flag-DGCR8-WT or -K^707^R were used for immunoprecipitation with anti-Flag antibody and then immunoblotting with anti-Drosha antibody. One-tenth of lysates as input were analyzed by immunoblotting. (**B**) There is no significant difference in the expression level of mature miRNA between low and high levels of DGCR8 SUMOylation. Pri-miR130b and Flag-DGCR8 with or without His-SUMO1 plasmids were transfected into stable cell lines 293T-shControl or -shSenp1. Forty-eight hours after transfection, half of the cells were used for extraction of total RNA, while the others were lysed for the SUMOylation assay with Ni^2+^-NTA resin. The expression levels of mature miR130b were analyzed by qRT-PCR (left panel) and the SUMOylation levels of DGCR8 were determined by western blotting (right panel). (**C** and **D**) SUMOylation of DGCR8 does not affect the microprocessor activity. The microprocessor reporter psiCHECK-pri-miR130b with control vecter, or DGCR8-WT or DGCR8-K^707^R were transfected into 293T (C) or HeLa-shDGCR8 (**D**), then 48 h after transfection cells were harvested for the dual-luciferase reporter assay.

As it is well known, DGCR8 is the most important partner of Drosha and combines with pri-miRNAs through its dsRBDS, thus helping Drosha cleave pri-miRNAs to pre-miRNAs. Therefore, to test whether K^707^-SUMOylation of DGCR8 alters its association with Drosha, we transfected Flag-DGCR8-WT or Flag-DGCR8-K^707^R into 293T or HeLa cells, respectively, and performed co-immunoprecipitation with anti-Flag antibody and followed by immunoblotting with anti-Drosha. The results showed that the SUMO-site mutation K^707^R of DGCR8 did not change the interaction between DGCR8 and Drosha (Figure 4A), indicating that K^707^-SUMOylation of DGCR8 might not influence miRNA biogenesis. To verify this, we investigated the expression levels of miRNAs in five cell lines A549, LM7, PC3, P69 and M12 stably expressing DGCR8-WT or DGCR8-K^707^R by Q-PCR. Indeed, these miRNAs including let-7a, miR-125a-5p, miR-125b, miR-138 and miR-146a displayed little difference between DGCR8-WT and DGCR8-K^707^R in above stable cell lines (Supplementary Figure S4A). Furthermore, we transfected DGCR8 with SUMO1 plasmid into the Senp1-knockdown 293T cells, in which DGCR8 was highly SUMOylated (Figure 4B, right panel, lane 6). However, the expression levels of mature miR-130b (as a representative) had no significant difference between low and high levels of DGCR8 SUMOylation (Figure 4B, left panel). Thus, these data suggest that DGCR8 SUMOylation slightly affects miRNA biogenesis.

Based on the above data and the classical miRNA biogenesis pathway, we wondered whether the SUMO1 modification at K^707^ of DGCR8 exerts no effect on the activity of MC either. To end this, we cloned the pri-miR-130b (containing pre-miR130b sequence together with the flanking upstream and downstream) into the 3′-UTR of Renilla luciferase gene in the psiCHECK2 vector (Supplementary Figure S4B) according to the construction method of MC reporter (28). To assess the effectiveness of this MC activation system, we transfected the above construct psiCHECK-pri-miR130b with DGCR8 and Drosha into 293T cells. As expected, compared to the control vector, the MC reporter activity (Firefly/Renilla) was significantly increased with DGCR8 alone, and more highly enhanced with both DGCR8 and Drosha (Supplementary Figure S4C). By using this system we found that there was no difference between DGCR8-WT and DGCR8-K^707^R on the microprocessor activities in 293T cells (Figure 4C). Then we re-expressed exogenous DGCR8-WT or DGCR8-K^707^R with the MC reporter in the stable HeLa-shDGCR8 cell line, and showed similar results that the SUMO-site mutation K^707^R did not affect the microprocessor activities (Figure 4D). Taken together, our data demonstrate that SUMOylation at K^707^ of DGCR8 slightly affects miRNA biogenesis, which is in a certain extent attributed to its having no effect on the microprocessor activity.

### SUMOylation of DGCR8 is required for the direct silencing effect of pri-miRNA by increasing its affinity with pri-miRNA

**Figure 5.**
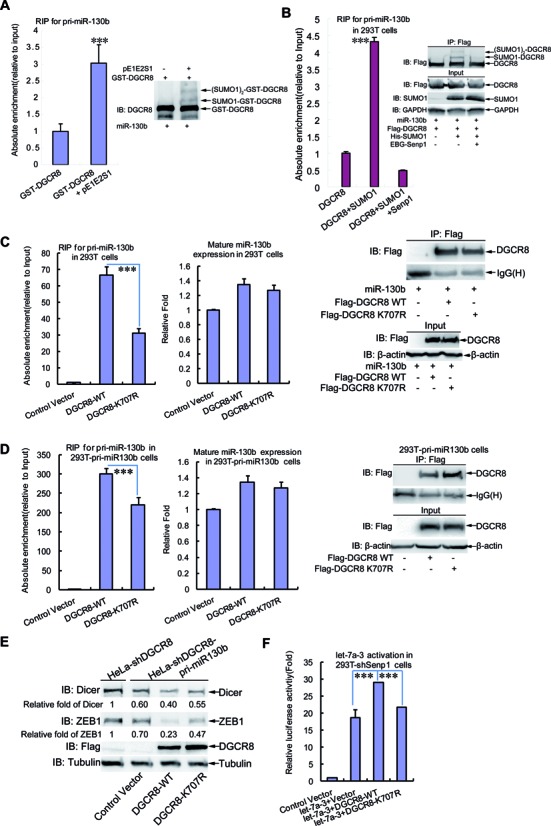
SUMOylation of DGCR8 increases its affinity with pri-miRNA and silencing effect. (**A**) SUMOylation of GST-DGCR8 in *Escherichia coli* enhances its recruiting pri-miRNA. GST-DGCR8 with or without pE1E2S1 expressed in *E. coli* was purified for RNA pull-down assay. Lysates from 293T cells transfected with pri-miR-130b was added to the same amount of above GST-proteins and GST-beads. After incubation and washing, one-tenth of the combination was subjected to western blotting, while nine-tenth was treated with Trizol for RNA purification and followed by qRT-PCR for pri-miR130b. The SUMO1 modification level of GST-DGCR8 was determined (right panel) and the relative recruitment of pri-miR130b by GST-DGCR8 was normalized with total pri-miR-130b in 293T cells (left panel). (**B**) Association with pri-miR-130b of DGCR8 is enhanced by its SUMO1 modification. Lysates from 293T cells co-transfected with pri-miR-130b, Flag-DGCR8, His-SUMO1 with or without Senp1 were used for RIP assay with anti-Flag antibody. After incubation and washing, one-tenth of the combination was subjected to western blotting, while 9/10 was treated with Trizol for RNA purification and followed by qRT-PCR for pri-miR130b. The relative recruitment of pri-miR130b by DGCR8 in RIP was normalized with total pri-miR-130b in 293T cells (left panel), and the SUMOylation level and IP efficiency were assessed by western blotting (right panel). (**C** and **D**) K^707^-SUMOylation of DGCR8 influences its binding with pri-miRNA. Lysates from (C) 293T cells co-transfected with pri-miR-130b and Flag-DGCR8-WT or -K^707^R, or from (D) 293T-pri-miR130b cells transfected with Flag-DGCR8-WT or -K^707^R, were immunoprecipitated with anti-Flag antibody and then treated with Trizol followed by qRT-PCR for pri-miR130b. The relative recruitment of pri-miR130b by DGCR8 was calculated by normalizing pri-miR130b from the immunoprecipition to the Input group (left panel). The expression level of mature miR130b was analyzed by qRT-PCR (middle panel) and the immunoprecipitation was assessed by western blotting (right panel). (**E**) The efficiency of pri-miRNA direct function in silencing target gene mediated by DGCR8 is dependent on its K^707^-SUMOylation. The stable cell line HeLa-shDGCR8-pri-miR130b was transfected with control vector, or DGCR8-WT or DGCR8-K^707^R for re-expression. HeLa-shDGCR8 cells were used as a control. Forty-eight hours after transfection, cells were lysed in SDS-lysis buffer for detection of Dicer and ZEB1 by western blotting. Quantification was analyzed by ImageJ (V1.45) and the Dicer or ZEB1 bands were normalized with the Tubulin bands. (**F**) K^707^-SUMOylation of DGCR8 regulates pri-let-7a-3 activation. The reporter construct psiCHECK-4LCS*let-7a3 and Flag-DGCR8-WT or -K^707^R with or without pri-let-7a-3 were transfected into 293T-shsenp1 cells, then 48 h after transfection cells were harvested for the dual-luciferase reporter assay.

To determine whether SUMOylation of DGCR8 influences its binding with pri-miRNAs, we performed an *in vitro* RNA-binding protein immunoprecipitation assay (RIP) with purified GST-DGCR8 from *E. coli* cells co-transformated with or without pE1E2S1. The recruitment of pri-miR-130b by GST-DGCR8 co-transformed with pE1E2S1 was increased about three-fold when compared with or without pE1E2S1 (Figure 5A, left panel), which was positively correlated with the SUMOylation level of GST-DGCR8 (Figure 5A, right panel). This strongly demonstrates that SUMOylation of DGCR8 can enhance its binding with pri-miRNA. Further, an RIP assay was performed with lysates from 293T cells co-transfected with pri-miR-130b, Flag-DGCR8, His-SUMO1 with or without Senp1. As expected, the binding of DGCR8 with pri-miR-130b was notably increased by SUMOylation of DGCR8 in the co-transfected with SUMO1 plasmid, whereas it was greatly reduced when SUMOylation of DGCR8 was removed by co-transfection of additional plasmid Senp1 (Figure 5B). In addition, we also confirmed that the affinity of DGCR8 binding with pri-miRNA in stable 293T-shSenp1 cells was significantly higher than that in 293T-shControl cells (Supplementary Figure S5A). Collectively, these results suggest that SUMOylation of DGCR8 enhances its affinity with pri-miRNA.

Next to determine whether K^707^-SUMOylation of DGCR8 is mainly responsible for influencing its binding with pri-miRNAs, we performed the RIP assay with the transient transfection of pri-miR-130b and DGCR8-WT or DGCR8-K^707^R into 293T cells. Indeed, the affinity of DGCR8 with pri-miR-130b was obviously decreased in mutant DGCR8-K^707^R compared to that in DGCR8-WT (Figure 5C, left panel), although the expression levels of mature miR-130b were scarcely changed among them (Figure 5C, middle panel). To further support this, we generated a stable 293T-pri-miR130b cell line expressing pri-miR-130b by the lentiviral vector system, and then repeated the RIP assay with this cell line showing the similar results (Figure 5D). Moreover, we also showed that the affinity of pri-miR-130b bound to the mutant DGCR8-E^709^A, which could effectively abolish SUMOylation on DGCR8-K^707^ (Supplementary Figure S5B), was greatly decreased as like that in DGCR8-K^707^R (Supplementary Figure S5C). This data strengthened the view that the reduced affinity with pri-miRNA of DGCR8-K^707^R was contributed to the decreased SUMOylation rather than the destroyed structure.

As observed that DGCR8-K^707^R did not affect the production of mature miR-130b, one puzzle emerged to us that what function and consequence were caused by the decreased affinity of DGCR8-K^707^R with pri-miR-130b. Nevertheless, it has already been reported that pri-miRNAs or pre-miRNAs can directly regulate their targets by themselves in addition to mature miRNAs (5–8,36), so we wondered whether the different affinities with pri-miR-130b between DGCR8-WT and DGCR8-K^707^R might bring about the different efficiency of gene silencing. To confirm this, we infected the lentiviral-system pri-miR-130b into HeLa-shDGCR8 cells to establish a stable cell line HeLa-shDGCR8-miR130b. Then we re-expressed exogenous DGCR8-WT or DGCR8-K^707^R in this cell line and determined the protein levels of DICER and ZEB1, both of which are targets of miR-130b (37,38). The data showed that protein levels of both DICER and ZEB1 in cells transfected with DGCR8-K^707^R were higher than those in cells transfected with DGCR8-WT (Figure 5E), which was in line with the results of the decreased affinity of DGCR8-K^707^R with pri-miR130b (Figure 5C and D). These results indicate that the efficiency of silencing target genes directly mediated by pri-miR-130b is dependent on K^707^-SUMOylation, which is required for the interaction between pri-miRNA and DGCR8.

To further validate that the attenuated affinity of pri-miRNA with DGCR8-K^707^R is relevant to the impaired gene silencing, we constructed a luciferase reporter construct psiCHECK2–4LCS*let-7a-3. Similar as the MC reporter (Supplementary Figure S4B), the four-repeat-tandem sequences complementary to let-7a-3 seeding sequence (4LCS) were inserted in the 3′-UTR of Renilla luciferase on the psiCHECK2 vector (Supplementary Figure S5D). 293T-shSenp1 cells were transfected psiCHECK2–4LCS*let-7a-3 and pri-let-7a-3 together with or without DGCR8-WT or DGCR8-K^707^R plasmids. Compared to the group transfected with psiCHECK2–4LCS*let-7a-3 alone, the let-7a-3 activation activity (firefly/renilla) was about 19- or 29-fold higher in groups co-transfected with pri-let-7a-3 alone or pri-let-7a-3 and DGCR8-WT together, respectively. However, when DGCR8-WT was substituted by DGCR8-K^707^R, the activity was fell back to the similar level as pri-let-7a-3 alone (Figure 5F). These data indicate that the pri-let-7a-3 activation mediated by DGCR8 is at least in partially regulated by its K^707^-SUMOylation.

### SUMOylation at K^707^ of DGCR8 promotes tumorigenesis and tumor cell migration

**Figure 6.**
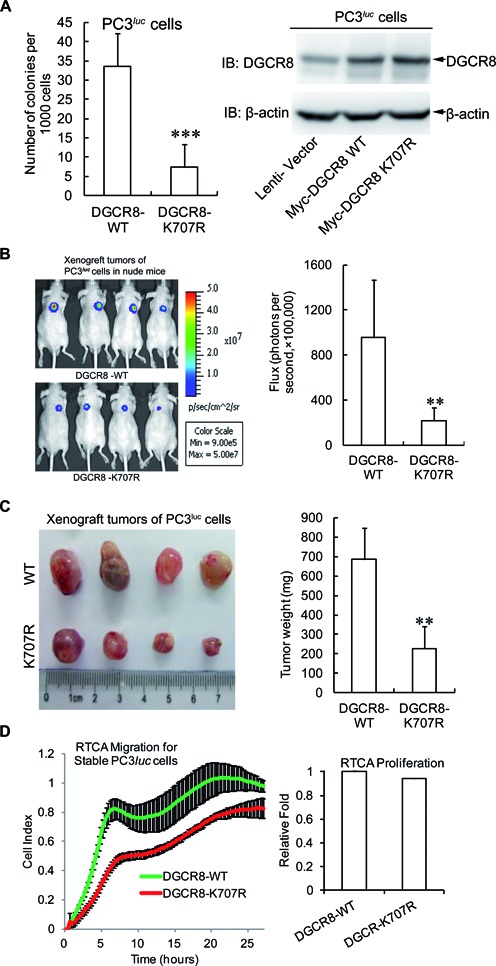
SUMOylation at K^707^ of DGCR8 promotes tumorigenesis and tumor cell migration. (**A**) DGCR8-K^707^R reduces the colony formation of PC3*^luc^* cells. Each of 1.5 × 10^3^ cells stably expressing DGCR8-WT or DGCR8-K^707^R were seeded in 2 ml of medium containing 1% FBS with 0.35% agar and layered onto the base with 0.6% agar. The colonies were stained with 0.005% crystal violet at day 21, and then photographs were taken and the number of colonies was scored by ImageJ V1.45 (NIH, USA). Three independent experiments were performed in triplicate. (**B** and **C**) DGCR8-K^707^R suppresses tumor growth in nude mice. (B) Backs of 6-week-old nude mice were subcutaneously injected with 2.5 × 10^6^ PC3*^luc^* cells stably expressing DGCR8-WT or -K^707^R. After injection for 14 days, tumor was assessed by bioluminescent imaging with a Xenogen IVIS imaging system and the tumor bioluminescent flux was quantified. (C) All mice were sacrificed at 30 days and tumors were dissected, photographed and weighted. (**D**) SUMOylation of DGCR8 affects tumor cell migration. For RTCA-Migration assay, 2 × 10^4^ of PC3*^luc^* cells stably expressing DGCR8-WT or -K^707^R were seeded into the upper chambers of the CIM-plate, and normal growth medium containing 10% FBS was added into the lower chamber. The kinetic cell indexes of their migration were recorded every 15 min (Left panel). For RTCA-proliferation assay, 2 × 10^3^ of cells were seeded into the E-Plate16. The real-time recording of proliferation was carried out on the RTCA-DP instrument (Roche) and monitored every 1 h for 3 days. The relative slope value of cell proliferation was calculated according to the instrument's instruction (right panel).

As DGCR8 is abnormally expressed in diverse cancers (14,16–19,39) and phosphorylation of DGCR8 can promote cell growth and migration (20), we wondered whether K^707^-SUMOylation of DGCR8 is connected with tumorigenesis and tumor cell migration. Since above data showed that DGCR8 K^707^ is a major SUMO1 modification site of DGCR8, we wanted to explore whether there is any functional difference between DGCR8-WT and -K^707^R. Therefore, we firstly compared the ability of anchor-independent growth of PC3*^luc^* (23) or A549*^luc^* cell lines stably expressing DGCR8-WT or -K^707^R, of which the expression level was comparable (Figure 6A and Supplementary Figure S6A, right panels). The results revealed that the numbers of colonies produced by PC3*^luc^* or A549*^luc^* cells transfected with DGCR8-K^707^R were substantially decreased compared to those in the transfected with DGCR8-WT (Figure 6A and Supplementary Figure S6A, left panels). These results indicate that DGCR8 SUMOylation potentially affects tumorigenesis. To further support this concept, each of above stable PC3*^luc^* or A549*^luc^* cell lines was inoculated subcutaneously into the backs of nude mice. At 14 days after injection tumors were assessed by bioluminescent imaging with a Xenogen IVIS imaging system (29,40). As shown in Figure 6B, tumors in the PC3*^luc^* group transfected with DGCR8-K^707^R grew more slowly than those in the DGCR8-WT group, which was consistent with the results of the colony formation assays. The average sizes and weights of tumors in the DGCR8-K^707^R group were also significantly reduced compared to those in the DGCR8-WT group at 30 days after injection (Figure 6C). Moreover, tumor growth of A549*^luc^* group transfected with DGCR8-K^707^R was almost completely inhibited whereas the DGCR8-WT group grew normally (Supplementary Figure S6B and C). Therefore, above data demonstrated that K^707^-SUMOylation of DGCR8 is crucial for tumorigenesis.

To test whether SUMOylation of DGCR8 influences the migration ability of tumor cell as well, we evaluated the cell motility by RTCA (26,29). The SUMO-site mutant DGCR8-K^707^R obviously decreased the capability of tumor cell migration compared to those of DGCR8-WT in both PC3*^luc^* and A549*^luc^* cell lines (Figure 6D and Supplementary Figure S6D, left panels). This was not resulted from cell proliferations, which had almost no differences between DGCR8-WT and - K^707^R in both cell lines (Figure 6D and Supplementary Figure S6D, right panels). Thus, our data reveal that K^707^ -SUMOylation of DGCR8 is linked to its new functions in regulation of tumorigenesis and tumor cell migration.

## DISCUSSION

**Figure 7.**
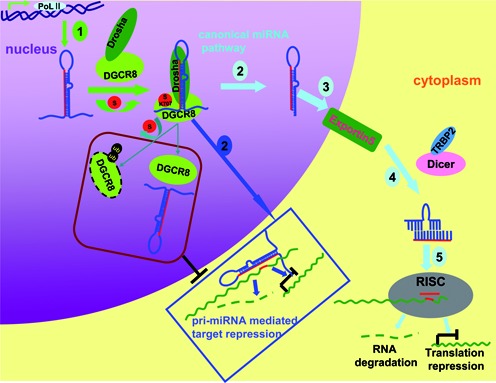
A model for DGCR8 SUMOylation controlling of pri-miRNA direct function. Generally, miRNAs are firstly transcribed by RNA ploymerase II termed as pri-miRNAs, then it is cleaved by Drosha-DGCR8 Microprocessor complex to pre-miRNAs in nucleus. Pre-miRNNAs are transported by Exportin5 to cytoplasm, where the second cleavages are undertaken by Dicer to form a duplex. Finally, the mature miRNA is loaded into the RISC complexes and performs the gene silencing by degradation of the target mRNA or repressing its translation. Apart from as a biogenesis intermediate only, pri-miRNAs exercise direct functions in recognition and repression of the target mRNAs (marked with dark blue Box). DGCR8 SUMOylation at K^707^ increases its protein stability by preventing the degradation via the ubiquitin proteasome pathway, and its affinity with pri-miRNA thus positively promoting the pri-miRNA direct recognition and repression of the targeted mRNA.

DGCR8, a double-stranded RNA binding protein, acting as the major partner of Drosha in MC of the miRNA biogenesis pathway has been intensively investigated on the basis of DGCR8 deficiency (41–43). The expression of DGCR8 is tightly controlled in organism because it is required for normal miRNA biogenesis and physiological functions. Deregulations of DGCR8 expression associating with the aberrant expressions of miRNAs have also been detected in many diseases such as schizophrenia (44–46) and different kinds of cancers (13,15,16,42,47). Recently, some modifications of DGCR8 affecting its stability and function have been paid attention. Deacetylation by HDAC1 on the key lysines in the dsRBDs of DGCR8 increases the affinity of DGCR8 with pri-miRNAs, consequently enhancing the miRNA processing (21). At least 23 phosphorylation sites on full-length human DGCR8 are mapped, and phosphorylation of DGCR8 by ERK/MAPK increases its stability but does not influence specific processing activity (20). Here we found that DGCR8 was modified by SUMO1 at the major site K^707^ and this modification was promoted by its ERK-activated phosphorylation and further enhanced its protein stability (Figures 1–3). SUMOylation of DGCR8 did not alter its association with Drosha, or miRNA biogenesis, but rather affected the affinity with pri-miRNAs to control the direct function of pri-miRNAs in target repression, which was linked to tumorigenesis and tumor cell migration. Our findings are summarized in Figure 7.

### Cross-talks among SUMOylation, phosphorylation and ubiquitination of DGCR8

We observed that DGCR8 protein was accumulated after the treatment with MG132 (Figure 3C), suggesting that DGCR8 degradation mainly depends on the ubiquitin proteasomal pathway. There were some interactions and cross-talks among ubiquitination, SUMOylation and phosphorylation of DGCR8. Firstly, SUMOylation of DGCR8 was promoted by its ERK/MAPK-mediated phosphorylation (Figures 1E, F and 3E), which probably altered the structure of DGCR8 in favor of SUMOylation occurring. Secondly, ERK-mediated phosphorylation (20) and K^707^-SUMOylation could synergistically stabilize DGCR8 by preventing from degradation of DGCR8 (Figure 3A, B, E and F, Supplementary Figure S3A). Thirdly, ubiquitination of DGCR8 was reduced by phosphorylation-promoted K^707^-SUMOylation (Figure 3D–F). Collectively, our findings suggest a mechanism by which phosphorylation, SUMOylation and ubiquitination cooperatively control the stability of DGCR8. However, the detailed mechanism how SUMO1 modification to block DGCR8 ubiquitination is unclear and seems worth investigating further.

### SUMOylation of DGCR8 regulates the direct silencing effects of pri-miRNA

SUMOylation of DGCR8 appeared not to influence miRNA biogenesis (Figure 4B, Supplementary Figure S4A). Consistently with this, the microprocessor activity was also not affected by this modification (Figure 4C and D). These could be reasonably interpreted with the results that K^707^-SUMOylated DGCR8 did not alter its interaction with Drosha (Figure 4A), and also the interaction region of DGCR8 with Drosha is located in the region of amino acids 738–750 (4) (Figure 4A). K^707^ is not resided in the two dsRBDs ranging from residues 511–576 and 620–684, respectively, but it is located at the terminal tail of the helix 5 (H5) that is tightly packed against both dsRBDS to form a compact overall structure (48). Thus, we speculated that K^707^-SUMOylation of DGCR8 increasing the affinity of DGCR8 with pri-miRNAs (Figure 5A–D, Supplementary Figures S5A and S5C) was possibly attributed to its altering the protein interacting surfaces.

Recently Dr Chen's group proposed a new concept that mature miRNAs are not the only target-recognizing species produced from miRNA genes, and pri-/pre-miRNAs also play roles in target recognition and repression (5–7,36). Kay's group also presented the evidences to strongly support the hypothesis that miRNA precursors are not mere biogenesis intermediates but also as direct regulators of miRNA activity (8). In this study we showed that the different repression levels of DICER and ZEB1 in cells transfected with DGCR8-WT and DGCR8-K^707^R were contributed to their different effects on the direct gene silencing of pri-miR-130b (Figure 5E), as there were changes in the affinity of DGCR8 with pri-miR-130b other than in the expression levels of mature miR-130b (Figure 5C and D). Moreover, we also provided evidences that the pri-let-7a-3 activation was regulated by K^707^-SUMOylation of DGCR8 (Figure 5F), which increased the binding of DGCR8 with pri-let-7a-3 thus promoting the direct recognition and repression of the targeted sequences of pri-let-7a-3.

### SUMOylation of DGCR8 is involved in tumorigenesis

It is known that SUMOylation is linked with diseases such as cancer. We found that the SUMO-site mutation of DGCR8-K^707^R inhibited the anchor-independent growth, xenograft tumor growth and tumor cell migration (Figure 6, Supplementary Figure S6), and SUMOylation of DGCR8 enhanced its affinity with pri-miRNA as well as the direct repression of target by pri-miRNA. Therefore, the functional differences between DGCR8-WT and -K^707^R on tumorigenesis were probably contributed to the different affinity of DGCR8 with some pri-miRNAs that were involved in tumorigenesis. Given that DGCR8 can interact with different kinds of RNAs and endonucleases such as hundreds of mRNAs, snoRNAs and lncRNAs (49) besides miRNA and Drosha, it is still unknown whether K^707^-SUMOylation influences the association of DGCR8 with them. It might be agreed that SUMOylation of DGCR8 also affects its interactions with many other unknown RNAs and proteins that are involved in tumorigenesis and cell migration.

## Supplementary Material

SUPPLEMENTARY DATA
