# Supplementary material for: SUMOylation at K707 of DGCR8 controls direct function of primary microRNA
Source: Nucleic Acids Res. 2015 Jul 21;43(16):7945–60. doi: 10.1093/nar/gkv741 (PMC4652762; doi:10.1093/nar/gkv741)
Supplement: SUPPLEMENTARY DATA [file supp_43_16_7945__index.html]

SUMOylation at K707 of DGCR8 controls direct function of primary microRNA — SUMOylation at K707 of DGCR8 controls direct function of primary microRNA — SUPPLEMENTARY DATA 

# SUMOylation at K707 of DGCR8 controls direct function of primary microRNA

## SUPPLEMENTARY DATA

- SUPPLEMENTARY DATA
- SUPPLEMENTARY DATA
- SUPPLEMENTARY DATA
- SUPPLEMENTARY DATA
- SUPPLEMENTARY DATA
- SUPPLEMENTARY DATA
- SUPPLEMENTARY DATA
- SUPPLEMENTARY DATA
