# Supplementary material for: SUMOylation at K707 of DGCR8 controls direct function of primary microRNA
Source: Nucleic Acids Res. 2015 Jul 21;43(16):7945–60. doi: 10.1093/nar/gkv741 (PMC4652762; doi:10.1093/nar/gkv741)
Supplement: SUPPLEMENTARY DATA [file supp_gkv741_nar-00407-y-2015-File009.doc]

**SUPPLEMENTARY FIGURE LEGEND**

**Figure S1.** (A) SUMOplot prediction of human DGCR8 protein**.** SUMOylation of human DGCR8 protein was predicated by the program of Abgent SUMOplot (<http://www.abgent.com/SUMOplot> ) based on the SUMOylation consensus sequence ΨKXE/D (where Ψ is a hydrophobic amino acid, K is the target lysine, X is any amino acid, D/E is an acid residue). K^707^ shows the second highest score(0.93). (B) SUMOylation of DGCR8 occurs naturally in 293T cells. 293T cells were lysed in NEM-RIPA buffer for immunoprecipitation with anti-DGCR8 or normal IgG, and then immunoblotted with anti-SUMO1 antibody. The same membrane was stripped for immunoblotting with anti-DGCR8 antibody. 1/10 of lysates as an input were immunoblotted.(C) The SUMOylation level of DGCR8 is decreased by mutation of phosphorylation sites. Phosphorylation sites of DGCR8 are mapped according to the previous study (20), and a key phosphorylation deficiency mutant Mut4, S109V/S153V/T371A/S377V, is shown. 293T cells co-tranfected with DGCR8-WT or DGCR8-Mut4 with His-SUMO1 were lysed for the SUMOylation assay with Ni^2+^-NTA resin.

**Figure S2.** K^707^ is a major SUMO1 modification site of DGCR8 in stable A549^luc^-DGCR8 cells. A549^luc^ cells stably expressing myc-DGCR8-WT or myc-DGCR8-K^707^R were lysed in NEM-RIPA buffer for immunoprecipitation with anti-Myc, and then immunoblotted with anti-SUMO1 antibody. The same membrane was stripped for immunoblotting with anti-SUMO2/3 antibody. 1/10 of lysates as an input were immunoblotted.

**Figure S3.** SUMOylation of DGCR8 enhances its protein stability. (A) Half-life of endogenous DGCR8 is prolonged by SUMOylation. HeLa cells transfected with/without HA-SUMO1 were treated with 100 μg/ml cycloheximide (CHX) as indicated time after transfection for 24 h. Then cells were lysed for immunoblotting analysis. **(B-C)** Expression of DGCR8 in HeLa-shDGCR8 cells. (**B**) Knockdown of DGCR8 in HeLa-shDGCR8 cells. HeLa cells was infected with lenti-viral shRNA for the 3’ untranslated region of DGCR8 or the Control shRNA. These stable cell lines were lysed in SDS-lysis buffer and subjected to Western blotting with anti-DGCR8 antibody for checking the knockdown efficiency. (**C**) Re-expression of DGCR8-WT and DGCR8 K^707^R in HeLa-shDGCR8 cells. HeLa-shDGCR8 cells transfected with Flag-DGCR8-WT or Flag-DGCR8-K^707^R were lysed for immunoblotting detection of the expression levels of exogenous DGCR8. These are related to Figure 3B.

**Figure S4. SUMOylation at K^707^ of DGCR8 does not influence miRNA biogenesis.** (**A**) No change in the expression levels of mature miRNAs in various cell lines stably expressing DGCR8-WT or DGCR8-K^707^R. Five cell lines A549, LM7, PC3, P69, M12 stably expressing DGCR8-WT or DGCR8-K^707^R were lysed with Trizol and the expression levels of mature miRNAs were detected by qRT-PCR. This is related to Figure 4B. (**B-C**) Schematic representation of the Microprocessor activation system. The human pri-miR130b containing the stem-loop structure with the flanking upstream and down stream sequences was cloned to the 3’-UTR of Renilla luciferase gene in the psiCHECK2 plasmid. The activation effect was measured by Firely/Renilla (**B**). 293T cells were transfected with psiCHECK-pri-miR130b with DGCR8 alone or together with Drosha. 48 h after transfection cells were harvested for the Dual-luciferase reporter assay(**C**). This is related to Figure 4C.

**Figure S5.** DGCR8-K^707^R reduces its affinity with pri-miRNA. (**A**) RIP assay in 293T-shControl and 293T-shSenp1 cells. Lysates from 293T-shControl or 293T-shSenp1 cells transfected with pri-miR-130b and Flag-DGCR8 were used for RIP assay with anti-Flag antibody. After incubation and washing, 1/10 of the combination was subjected to Western blotting, while 9/10 was treated with Trizol for RNA purification and followed by qRT-PCR for pri-miR130b. The relative recruitment of pri-miR130b by DGCR8 in RIP was normalized with total pri-miR-130b in cells (left panel), and IP efficiency were assessed by Western blotting (right panel). (**B**) SUMO1 modification of DGCR8 is abolished by the point mutation E^709^A. 293T cells co-tranfected with DGCR8-WT, -K^707^R or -E^709^A, and His-SUMO1 were lysed for the SUMOylation assay with Ni^2+^-NTA resin. (**C**) SUMOylation at K^707^ of DGCR8 influences its binding with pri-miRNA. Lysates from 293T cells co-transfected Flag-DGCR8-WT, -K^707^R or -E^709^A and pri-miR-130b were used RIP assay. The relative recruitment of pri-miR130b by DGCR8 (Left panel) and IP efficiency (right panel) were analyzed as above. (**D**) Schematic representation of the let7a-3 activation system. Tetra-tandem sequences complementary to the seeding sequences of human let-7a-3 were cloned to the 3’-UTR of Renilla luciferase gene in the psiCHECK2 vector. The activation effect with pri-let-7a-3 and Drosha/DGCR8 was calculated by Firely/Renilla. This is related to Figure 5F.

**Figure S6.** SUMOylation at K^707^ of DGCR8 Promotes Tumorigenesis and Tumor Cell Migration. (**A**) DGCR8-K^707^R reduces the colony formation of A549*^luc^* cells. Each of 2 × 10^3^ cells stably expressing DGCR8-WT or DGCR8-K^707^R were seeded in 2 ml of medium containing 1% FBS with 0.35% agar and layered onto the base with 0.6% agar. The colonies were stained with 0.005% crystal violet at day 14, and then photographs were taken and the number of colonies was scored by ImageJ V1.45 ( NIH , USA). Three independent experiments were performed in triplicate. (**B-C**) DGCR8-K^707^R suppresses tumor growth in nude mice. (**B**) Backs of 6-week-old nude mice were subcutaneously injected with 2.5×10^6^ A549*^luc^* cells stably expressing DGCR8-WT or -K^707^R. After injection for 14 days, tumor was assessed by bioluminescent imaging with a Xenogen IVIS imaging system. And the tumor bioluminescent flux was quantified. (**C**) All mice were sacrificed at 30 days and tumors were dissected, photographed and weighted. (**D**) SUMOylation of DGCR8 affects tumor cell migration. For RTCA-Migration assay, 2 × 10^4^ of PC3*^luc^* cells stably expressing DGCR8-WT or -K^707^R were seeded into the upper chambers of the CIM-plate, and normal growth medium containing 10% FBS was added into the lower chamber. The kinetic cell indexes of their migration were recorded every 15 min (Left panel). For RTCA-Proliferation assay, 2 × 10^3^ of cells were seeded into the E-Plate16. The real-time recording of proliferation was carried out on the RTCA-DP instrument (Roche) and monitored every 1 h for 3 days. The relative slope value of cell proliferation was calculated according to the instrument’s instruction (right panel).
