# Supplementary material for: SUMOylation at K707 of DGCR8 controls direct function of primary microRNA
Source: Nucleic Acids Res. 2015 Jul 21;43(16):7945–60. doi: 10.1093/nar/gkv741 (PMC4652762; doi:10.1093/nar/gkv741)

Figure S1

A SUMOplot prediction of human DGCR8

**Protein ID:**	gi|38488720|ref|NP_073557.3|
**Defintion:**	microprocessor complex subunit DGCR8 isoform 1 [Homo sapiens]
**Length:**	773 aa

```
  1 METDESPSPL PCGPAGEAVM ESRARPFQAL PREQSPPPPL QTSSGAEVMD
 51 VGSGGDGQSE LPAEDPFNFY GASLLSKGSF SKGRLLIDPN CSGHSPRTAR
101 HAPAVRKFSP DLKLLKDVKI SVSFTESCRS KDRKVLYTGA ERDVRAECGL
151 LLSPVSGDVH ACPFGGSVGD GVGIGGESAD KKDEENELDQ EKRVEYAVLD
201 ELEDFTDNLE LDEEGAGGFT AKAIVQRDRV DEEALNFPYE DDFDNDVDAL
251 LEEGLCAPKK RRTEEKYGGD SDHPSDGETS VQPMMTKIKT VLKSRGRPPT
301 EPLPDGWIMT FHNSGVPVYL HRESRVVTWS RPYFLGTGSI RKHDPPLSSI
351 PCLHYKKMKD NEEREQSSDL TPSGDVSPVK PLSRSAELEF PLDEPDSMGA
401 DPGPPDEKDP LGAEAAPGAL GQVKAKVEVC KDESVDLEEF RSYLEKRFDF
451 EQVTVKKFRT WAERRQFNRE MKRKQAESER PILPANQKLI TLSVQDAPTK
501 KEFVINPNGK SEVCILHEYM QRVLKVRPVY NFFECENPSE PFGASVTIDG
551 VTYGSGTASS KKLAKNKAAR ATLEILIPDF VKQTSEEKPK DSEELEYFNH
601 ISIEDSRVYE LTSKAGLLSP YQILHECLKR NHGMGDTSIK FEVVPGKNQK
651 SEYVMACGKH TVRGWCKNKR VGKQLASQKI LQLLHPHVKN WGSLLRMYGR
701 ESSKMVKQET SDKSVIELQQ YAKKNKPNLH ILSKLQEEMK RLAEEREETR
751 KKPKMSIVAS AQPGGEPLCT VDV
```

Motifs with high probability

Motifs with low probability

Overlapping Motifs

No.	Pos.	Group	Score	No.	Pos.	Group	Score
1	K640	MGDTS **IKFE** VVPGK	0.94	8	K182	GESAD **KKDE** ENELD	0.48
2	K707	ESSKM **VKQE** TSDKS	0.93	9	K342	GTGSI **RKHD** PPLSS	0.44
3	K426	LGQVK **AKVE** VCKDE	0.79	10	K408	PGPPD **EKDP** LGAEA	0.39
4	K510	VINPN **GKSE** VCILH	0.67	11	K751	EREET **RKKP** KMSIV	0.34
5	K456	FEQVT **VKKF** RTWAE	0.58	12	K266	KRRTE **EKYG** GDSDH	0.33
6	K650	VPGKN **QKSE** YVMAC	0.5	13	K446	FRSYL **EKRF** DFEQV	0.15
7	K181	GGESA **DKKD** EENEL	0.5	14	K501	QDAPT **KKEF** VINPN	0.13

B

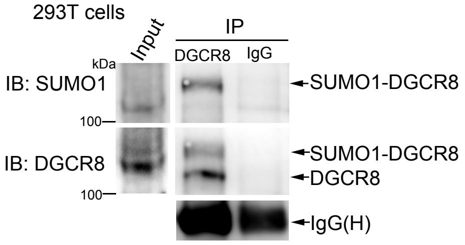

C

DGCR8

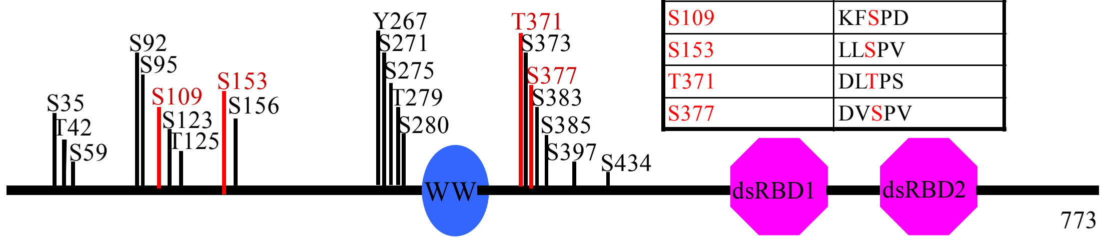

Phosph-site positions	Sequences
S109	KFSPD
S153	LLSPV
T371	DLTPS
S377	DVSPV

Mut4:S109A/S153V/T371A/S377V

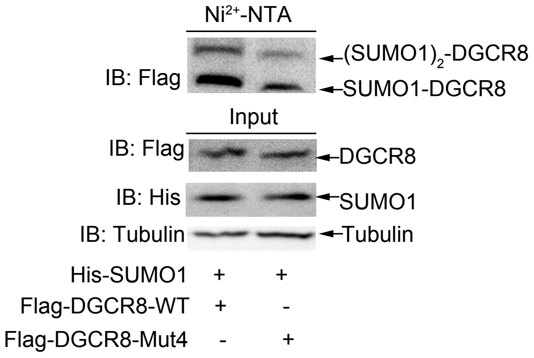

Supplement: SUPPLEMENTARY DATA [file supp_gkv741_nar-00407-y-2015-File010.pdf]
