# Supplementary figures and images for: SUMOylation at K707 of DGCR8 controls direct function of primary microRNA
Source: Nucleic Acids Res. 2015 Jul 21;43(16):7945–60. doi: 10.1093/nar/gkv741 (PMC4652762; doi:10.1093/nar/gkv741)

Figure S2

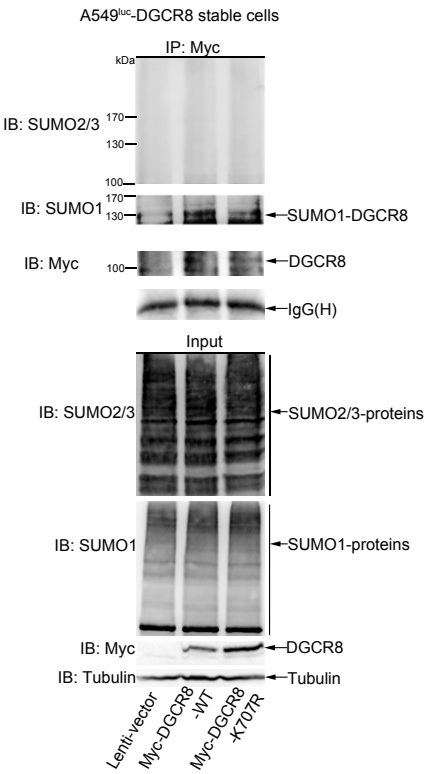

Supplement: SUPPLEMENTARY DATA [file supp_gkv741_nar-00407-y-2015-File011.pdf]

Figure S3

A

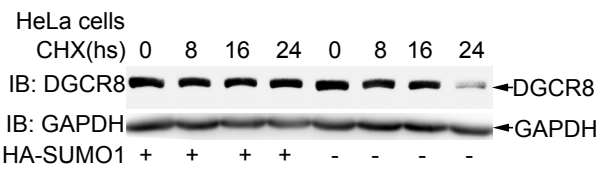

B

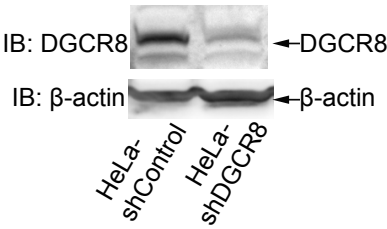

C

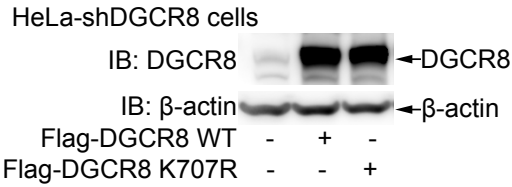

Supplement: SUPPLEMENTARY DATA [file supp_gkv741_nar-00407-y-2015-File012.pdf]

Figure S4

A

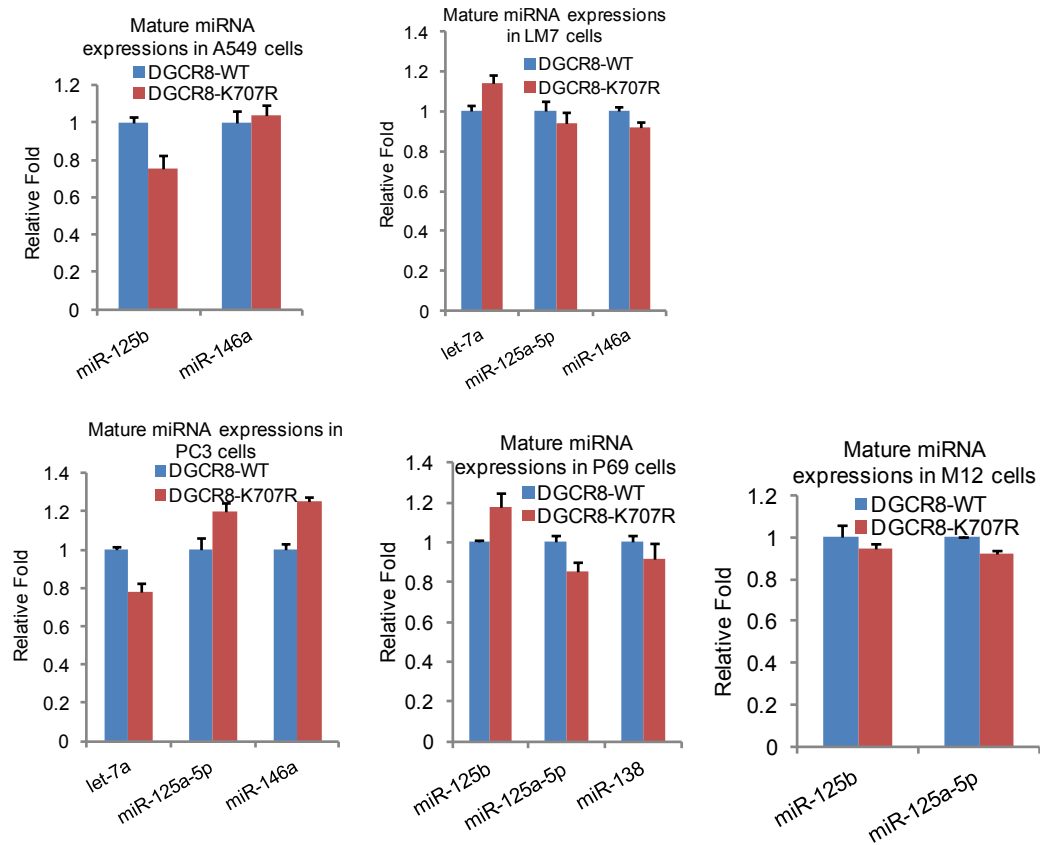

B

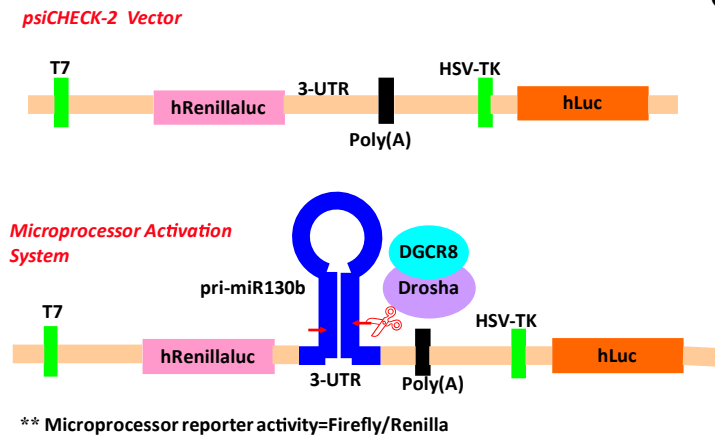

C

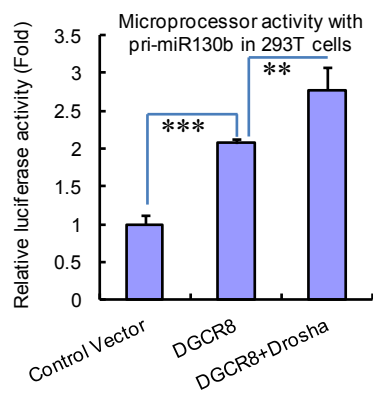

Supplement: SUPPLEMENTARY DATA [file supp_gkv741_nar-00407-y-2015-File013.pdf]

Figure S5

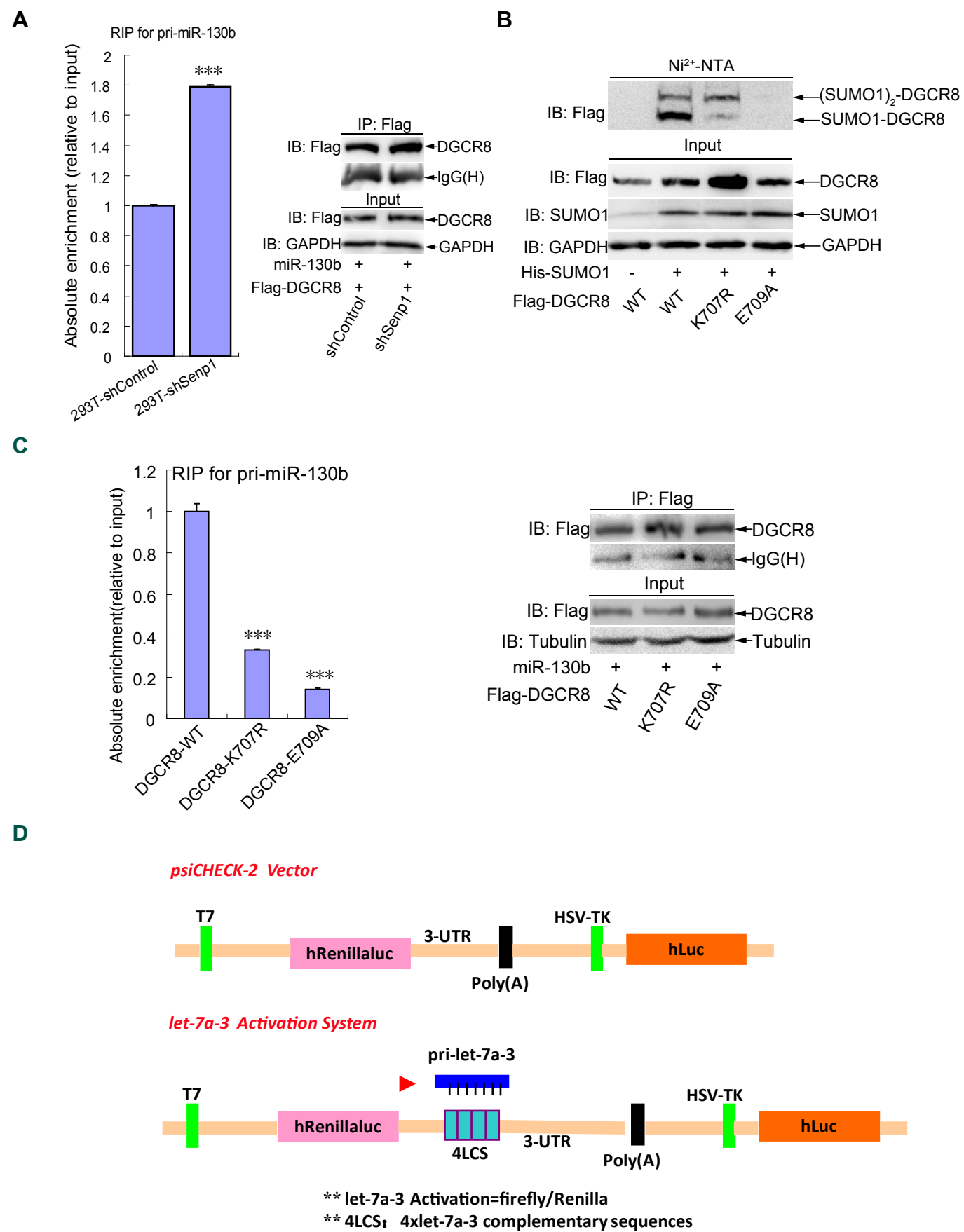

Supplement: SUPPLEMENTARY DATA [file supp_gkv741_nar-00407-y-2015-File014.pdf]

**Figure S6**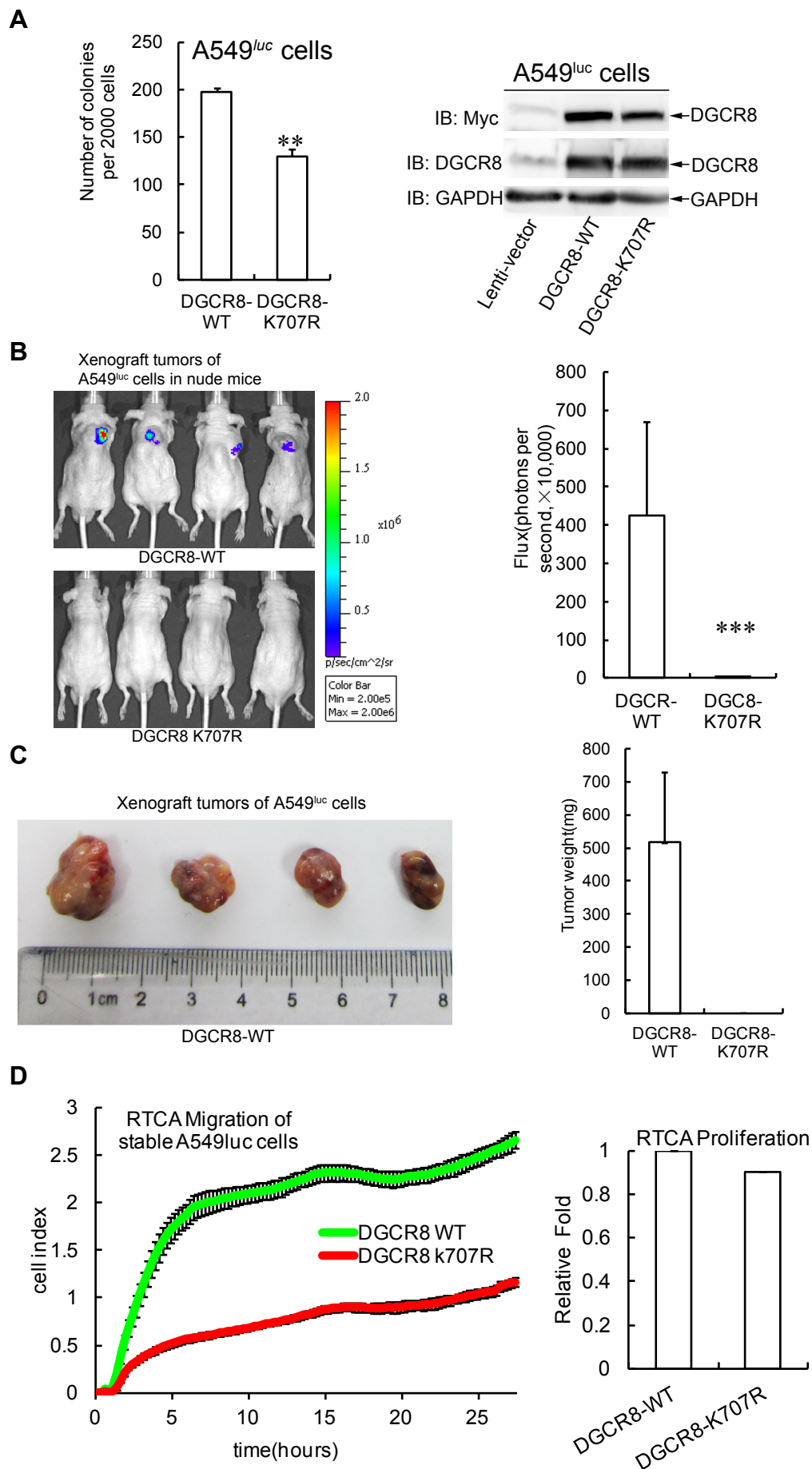

Supplement: SUPPLEMENTARY DATA [file supp_gkv741_nar-00407-y-2015-File015.pdf]
