# Supplementary material for: SUMOylation at K707 of DGCR8 controls direct function of primary microRNA
Source: Nucleic Acids Res. 2015 Jul 21;43(16):7945–60. doi: 10.1093/nar/gkv741 (PMC4652762; doi:10.1093/nar/gkv741)
Supplement: SUPPLEMENTARY DATA [file supp_gkv741_nar-00407-y-2015-File016.doc]

**Table S1. All primers or oligonucleotides used in this study.**

**1) Primers used for construction of** GST-DGCR8

| hDGCR8 | Primer | sequences (5’-3’) |
| --- | --- | --- |
| GST-DGC8 | Forward | CG*GAATTC*atggagacagatgagagccc |
|  | Reverse | GAAT*GCGGCCGC*tcacacgtccacggtgca |

**2) Primers for point mutation of DGCR8**

| hDGCR8 | Primer | sequence (5’-3’) |
| --- | --- | --- |
| DGCR8 K^181^R | Forward | AGGAAGGATGAGGAGAATGAGCTGG |
|  | Reverse | ATCAGCACTCTCACCCCCTATGCCT |
| DGCR8 K^426^R | Forward | AGAGTCGAGGTGTGCAAAGATGAAT |
|  | Reverse | GGCCTTCACCTGCCCCAGGGCCCCA |
| DGCR8 K^456^R | Forward | AGAAAATTCAGGACTTGGGCTGAGC |
|  | Reverse | CACAGTAACTTGCTCAAAGTCAAAA |
| DGCR8 K^510^R | Forward | AGATCCGAGGTCTGCATCCTGCACG |
|  | Reverse | CCCGTTGGGGTTAATAACAAACTCT |
| DGCR8 K^640^R | Forward | AGGTTTGAAGTGGTTCCTGGGAAAA |
|  | Reverse | GATAGACGTGTCACCCATCCCATGG |
| DGCR8 K^650^R | Forward | AGGAGTGAATACGTCATGGCGTGTG |
|  | Reverse | CTGGTTTTTCCCAGGAACCACTTCA |
| DGCR8 K^707^R | Forward | AGGCAGGAGACATCGGACAAGAGTG |
|  | Reverse | GACCATCTTGCTGCTCTCACGGCCA |
| DGCR8 E^709^A | Forward | CAGGCGACATCGGACAAGAGT |
|  | Reverse | CTTGACCATCTTGCTGCTCTC |
| DGCR8 S^109^V | Forward | CGGAAGTTCGCCCCTGACCTT |
|  | Reverse | GACCGCAGGTGCGTGCCG |
| DGCR8 S^153^V | Forward | CTTGTCCCTGTCAGTGGGGAC |
|  | Reverse | GAGCAGACCGCACTCCGCCCG |
| DGCR8 T^371^A/S^377^V | Forward | GCCCCTAGTGGGGATGTGGTC |
|  | Reverse | GAGGTCACTGCTTTGCTCCCG |

**3) Primers used for constructions of lentiviral DGCR8 expression, shRNAs for DGCR8 and Senp1**

|  | Primer | Sequence (5’-3’) |
| --- | --- | --- |
| lentiviral-DGCR8 | Forward | GC*TCTAGA*gccaccatggcatcaatgcaga |
|  | Reverse | GAAT*GCGGCCGC*tcacacgtccacggtgca |
| DGCR8-shRNA | Forward | *CCGGT*AGTCATGCATCGTGCACCACACTCG  AGTGTGGTGCACGATGCATGACTTTTTTTG |
|  | Reverse | *AATTC*AAAAAAAGTCATGCATCGTGCACCACA  CTCGAGTGTGGTGCACGATGCATGACTA |
| Senp1-shRNA | Forward | *CCGGT*AGAATACTCTTGCAATACCCTCGAGG  GTATTGCAAGAGTATTCTTTTTTG |
|  | Reverse | *AATTC*AAAAAAGAATACTCTTGCAATACCCTC  GAGGGTATTGCAAGAGTATTCTA |

**4) Primers for qRT-PCR**

| miRNAs | Primer | sequence (5’-3’) |
| --- | --- | --- |
| pri-miR-130b | Forward | tcagatccctgcagaccaccctg |
|  | Reverse | ggcagcaagctccctttcccc |
| GAPDH | Forward | atgaggtccaccaccctgtt |
|  | Reverse | ctcaagggcatcctgggcta |
| U6 | Forward | cgcttcggcagcacatatac |
|  | Reverse | aggggccatgctaatcttct |
| Universal primer | Reverse | gtgcagggtccgaggt |
| hsa-miR-130b | RT-primer | gtcgtatccagtgcagggtccgaggtattcgcactggatacgacatgccc |
|  | Forward | gcggcgcagtgcaatgatgaaa |
| hsa-miR-125b-1 | RT-primer | gtcgtatccagtgcagggtccgaggtattcgcactggatacgactcacaa |
|  | Forward | gcctatccctgagaccctaa |
| Let-7a-3 | RT-primer | gtcgtatccagtgcagggtccgaggtattcgcactggatacgacaacta |
|  | Forward | gccgctgaggtagtaggttgta |
| hsa-miR-146a | RT-primer | gtcgtatccagtgcagggtccgaggtattcgcactggatacgacaaccca |
|  | Forward | gctctagaaattctccatgttgcccag |
| hsa-miR-125a-5p | RT-primer | gtcgtatccagtgcagggtccgaggtattcgcactggatacgactcacag |
|  | Forward | gcctatccctgagacccttt |
| hsa-miR-138 | RT-primer | gtcgtatccagtgcagggtccgaggtattcgcactggatacgaccggcct |
|  | Forward | gatccgagctggtgttgtgaatc |

**5) Primers for construction psiCHECK**-pri-miR130b

| psiCHECK-pri-miR-130b | Forward | CC*CTCGAG*caatgatattgtcaaagca |
| --- | --- | --- |
|  | Reverse | AAATAT*GCGgccgc*tgctcctgcacctgtccag |

**6) The sequences below were inserted to psiCheck2 vector and the stem-loop sequence of pre-miR-130b is underlined:**

caatgatattgtcaaagcatctgggaccagccttggggatctccctccctataaccctcacctcccactccccaggcagggccccttgggcttgcaggccctcccctcccccctacccaattcgctcccttctccatggaaacttgagattctagacaggcctgggcctctggggtcagagggcaccctttccccccgggcagaggccccgccccagccagcctgcattccaggtctcagatccctgcagaccaccctgggggaggcactggcaGGCCTGCCCGACACTCTTTCCCTGTTGCACTACTATAGGCCGCTGGGAAGCAGTGCAATGATGAAAGGGCATCGGTCAGGTCcagcctgctaccctgggagggggaaagggagcttgctgcctcactccactttccagttgagaaggttgaggtgcccagagggtaagtgtttccactgattaggcacagatgtgaagctggactggaccctccatcagaggatcaggtgggtgcagagctggagaatcatccctaaactctccatcctgtcatggctggacaggtgcaggagca
